# Low-Threshold Optical Bistability in the Graphene-Oxide Integrated Asymmetric Nanocavity at Visible Light Frequencies

**DOI:** 10.3390/nano12071117

**Published:** 2022-03-28

**Authors:** Zeqiang Wang, Boyuan Cai, Zhengfen Wan, Yunyue Zhang, Xiaoguang Ma, Min Gu, Qiming Zhang

**Affiliations:** 1 Institute of Photonic Chips, University of Shanghai for Science and Technology, Shanghai 200093, China; 192380337@st.usst.edu.cn (Z.W.); caiboyuan@usst.edu.cn (B.C.); zhengfen.wan@usst.edu.cn (Z.W.); yunyuezhang@st.usst.edu.cn (Y.Z.); maxiaoguangkris@163.com (X.M.); 2 Centre for Artificial-Intelligence Nanophotonics, School of Optical-Electrical and Computer Engineering, University of Shanghai for Science and Technology, Shanghai 200093, China

**Keywords:** graphene oxide, nanocavity, optical bistability

## Abstract

Here, we propose an optical bistable device structure with a few layers of graphene oxide integrated in the metal-dielectric-metal based asymmetric nanocavity. Through the light confinement in the nanocavity, the third order nonlinear absorption of graphene oxide can be significantly enhanced, which experimentally delivers low-threshold optical bistability at the visible wavelength of 532 nm with only 267 KW/cm^2^ intensity. In addition, the switching threshold can be further reduced via increasing the graphene oxide thickness, hence paving a new way for achieving tunable optical bistable devices at visible light frequencies.

## 1. Introduction

Graphene oxide, a graphene-like oxidation two-dimensional (2D) material, has been investigated extensively because of its extraordinary optical nonlinear properties, which have been shown to be superior to normal dielectrics [1]. Usually, this large nonlinearity can lead to the required interaction length of light with the material reduced in many applications such as conductive transparent coatings [2,3], photocatalysts [4], microwave absorbers [5] and all-optical encryption [6]. However, GO material presents a significant linear loss, even though with a large nonlinear property. Therefore, to ensure sufficient signal reflection from the devices with enhanced optical nonlinearity, it is important to apply extra optical configurations to enlarge the interaction between GO and incident light in many applications, i.e., the optical bistable device [7].

Optical bistability (OB) means that an optical system has two possible output light intensity states under a given input light intensity, which realizes the recoverable switching transition between these two light intensity states [8,9]. Optical bistability has many potential applications in all-optical transistor [10] and all-optical memory [11]. However, most of the optical bistable device based on the solid-state materials usually have very high threshold (~GW/cm^2^) due to the low third-order susceptibility [12]. It is still a challenge to further reduce the threshold as much as possible for the further applications. Recently, graphene and its derivative, graphene oxide has attracted tremendous attention in the realization of optical bistable devices due to the outstanding nonlinear optical properties [13]. To further reduce the threshold of the device, delicate resonant configurations filled with nonlinear materials have been widely investigated mostly in the terahertz band [14], including photonic crystals [15], surface plasmons [16] and Fabry–Perot cavity [17]. For example, D. Zhao et al. theoretically studied the optical bistability in photonic multilayers doped by graphene sheets, stacking two Bragg reflectors with a defect graphene layer between the two Bragg reflectors. The reduced threshold of around 10 GW/cm^2^ can be obtained through utilizing the local field of defect mode to enhance the nonlinearity [18]. K.J. Ahn et al. theoretically investigated optical bistability of graphene inserted at the interface between two thin dielectric layers. Through optimizing the material properties and the thickness of the dielectric layers, optical bistable threshold of several MW/cm^2^ can be achieved [19]. Even though the graphene-based materials show large nonlinear susceptibility, the thresholds of these optical bistable devices are still quite high, usually in the MW/cm^2^ scale with the operation band limited in the terahertz frequency. Therefore, it is of great significance to design a device structure with the low threshold optical bistability especially in the KW/cm^2^ scale and the operation band at visible light frequencies [20,21].

In this paper, we proposed an optical bistable device with a few layers of graphene oxide integrated in the asymmetric nanocavity, which delivers low-threshold optical bistability at visible frequencies with only few hundreds of KW/cm^2^ laser power. The asymmetric nanocavity is based on metal-dielectric-metal (MDM) Fabry-Perot cavity with a plasmonic lossy ultrathin (~20 nm) metal film as the top layer. The multilayer graphene oxide (GO) is integrated in the dielectric layer by stacking 1 mg/mL solution of graphene oxide [22,23] and 2% solution of pdda for many times to achieve flat GO layers with the thickness control in few nanometers. Through coupling the light into the nanocavity from the ultrathin metal top layer, the nonlinear absorption of graphene oxide can be dramatically enhanced due to the cavity confinement effects on light waves, thereby reducing the optical bistability threshold of the device. The low-threshold optical bistability of 267 KW/cm^2^ at the wavelength of 532 nm can be achieved experimentally with six layers of graphene oxide (around 12 nm thick). We believe that both the large nonlinear response of graphene oxide and the strong field confinement in the asymmetric MDM nanocavity play the important roles in obtaining the low threshold optical bistability.

## 2. Simulation Models

**Figure 1:**
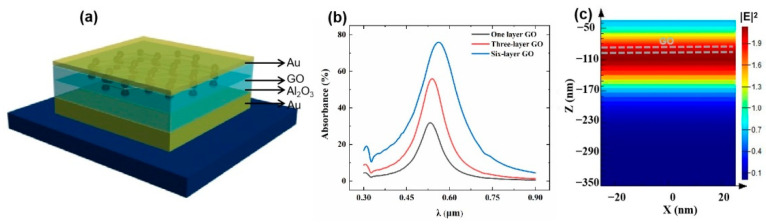
(**a**) The 3D schematic diagram of the GO integrated nanocavity. (**b**) The simulated absorption spectra of the device with different layers of GO by Lumerical FDTD. (**c**) The electric field distribution of the GO integrated cavity at the maximum absorption wavelength of 532 nm.

The three-dimensional schematic diagram of the GO integrated nanocavity structure considered in this work is shown in Figure 1a. The bottom metal layer is optically thick (120 nm) enough to maximize the light reflection of the device, while the top metal layer thickness is optimized (20 nm) for the balance between the light coupling from top surface and the strong light confinement in the nanocavity. A plane wave is incident on the nanocavity top surface. The cavity resonance wavelength can be tuned by changing the Al_2_O_3_ dielectric layer thickness through finite-difference time-domain (FDTD) simulation, covering the whole visible light wavelength range. To apply this asymmetric nanocavity for the nonlinear absorption enhancement of the GO, a 2 nm thick GO layer (corresponding to one layer of GO) is inserted in the middle of the nanocavity. The thickness of the upper and lower Al_2_O_3_ layer is optimized to be 40 nm and 45 nm, respectively, to achieve the cavity resonance around 532 nm in the green light wavelength region for the experimental device characterization with lasers in the following section. Figure 1b shows the plot of the simulated GO absorption in the designed nanocavity. It can be found that light absorption as high as 30% can be achieved with one layer of GO layer (2 nm thick), indicating that the threshold for the optical bistability of the device can be significantly reduced. With the layers of GO increasing to 6 layers (12 nm), over 75% light absorption can be achieved at the wavelength of around 550 nm. The electric field distribution of the GO integrated nanocavity is illustrated in Figure 1c to further demonstrate the light confinement mechanism. It can be seen that a standing wave can be obtained in the cavity due to the constructive interference of the in-coupling light and reflected light, leading to the absorption enhancement of GO due to the light confinement in the dielectric layer.

**Figure 2:**
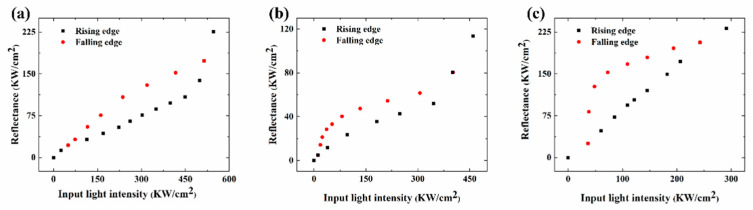
(**a**) Optical bistable hysteresis loop of the device integrated with one layer of GO by COMSOL simulation. (**b**) Optical bistable hysteresis loop of the device integrated with three-layer of GO by COMSOL simulation. (**c**) Optical bistable hysteresis loop of the device integrated with six-layer of GO by COMSOL simulation.

To simulate our cavity optical bistability behavior, the third-order nonlinear coefficients of the graphene oxide film need to be deduced first via fitting the measured curves by Z-scan system. Plus, then we build the optical bistability device model with the nonlinear parameters obtained by Z-scan for the COMSOL simulation. The wavelength is set to be 532 nm and the thicknesses of the GO layers are one layer, three layers and six layers for simulation. The optical bistability phenomenon of the three models can be achieved with the COMSOL simulation and the results of optical bistability hysteresis loops are shown in Figure 2. The optical bistable threshold of the nanocavity with one layer of graphene oxide is 514 KW/cm^2^ as shown in Figure 2a. The threshold can be reduced to 397 KW/cm^2^ for three layers of graphene oxide integrated nanocavity and for six-layer of graphene oxide, the optical bistable threshold of the device is as low as 243 KW/cm^2^ as shown in Figure 2b,c. It can be found that the switching threshold can be further reduced via increasing the graphene oxide thickness, which is due to absorption enhancement of the device with more layers of GO as illustrated in Figure 1b. We believe that the simulated optical bistability behavior of the device mainly originates from the enhanced nonlinear absorption of the GO films by light confinement effect in the asymmetric nanocavity.

## 3. Experimental Results and Discussion

**Figure 3:**
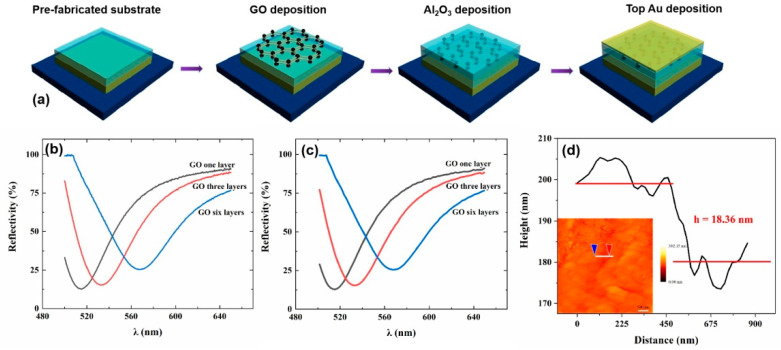
(**a**) The scheme of the GO integrated nanocavity fabrication process. (**b**) The measured reflection spectra for different layers of GO integrated nanocavity. (**c**) The simulated reflection spectra for different layers of GO integrated nanocavity. (**d**) The atomic force microscopy characterization of nine-layer graphene oxide.

To experimentally validate our device optical bistability performance, the GO integrated nanocavity device is fabricated as shown in Figure 3a. First, 120 nm thick gold film and 45 nm thick Al_2_O_3_ film were sequentially deposited on the polished wafer substrate by magnetron sputtering as a pre-fabricated substrate for the preparation of multilayer graphene oxide. Then we prepared 1 mg/mL solution of graphene oxide and put it into an ultrasonic cleaner to ultrasonically disperse it into suspension. The 20% solution of pdda was diluted to 2% solution and then the pre-fabricated substrate was soaked in 2% solution of pdda for one minute. After the pdda solution was evenly adsorbed on the substrate, the excess pdda solution was blow-dried with a nitrogen gun. Then the substrate with pdda was soaked in 1 mg/mL solution of graphene oxide for one minute, and after the anions and cations are attracted to each other, the graphene oxide can be evenly adsorbed on the pdda. The multilayer graphene oxide film can be obtained on the pre-fabricated substrate by repeating the above deposition steps as shown in Figure 3a. One, three and six-layer graphene oxide films were prepared in this experiment. Through atomic force microscopy (AFM) characterization as shown in Figure 3d, it can be found that the nine-layer GO film displays an overall thickness of around 18 nm, indicating that the thickness of one layer of GO film is around 2 nm. Finally, the 40 nm Al_2_O_3_ film and the 20 nm gold film were sputtered sequentially on the surface of the multilayer graphene oxide to form a complete nanocavity. The measured and simulated reflection spectra for the GO integrated nanocavity with different layers of GO are illustrated in Figure 3b,c. In Figure 3b,c, it can be found in the reflectance spectrum that a valley corresponds to the cavity resonance at the wavelength around 532 nm. The reflection valley shifts from around 532 nm to around 570 nm with the increase of GO layers from monolayer to six layers, indicating that the increase of the GO thickness results in a red-shift of the cavity resonance. According to our AFM characterization results as shown in Figure 3d, one layer of GO corresponds to 2 nm thickness film and the total thickness of the cavity can be increased from 97 nm to 107 nm with the layer of GO film increased to six. The red-shift of the reflection valley is mainly because of the varied resonance condition associated with the multiple round-trip phase shifts of electromagnetic wave inside the resonant cavity, which originates from the increase of the cavity thickness. Through the comparison of Figure 3b,c, the overall shape and resonance position of the experimental spectral features agree quite well with the simulated results.

**Figure 4:**
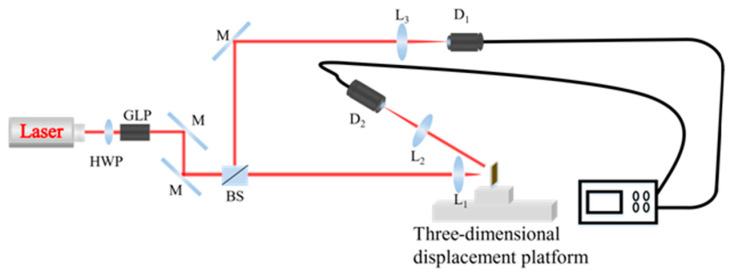
Optical path diagram of the optical bistability experiment.

**Figure 5:**
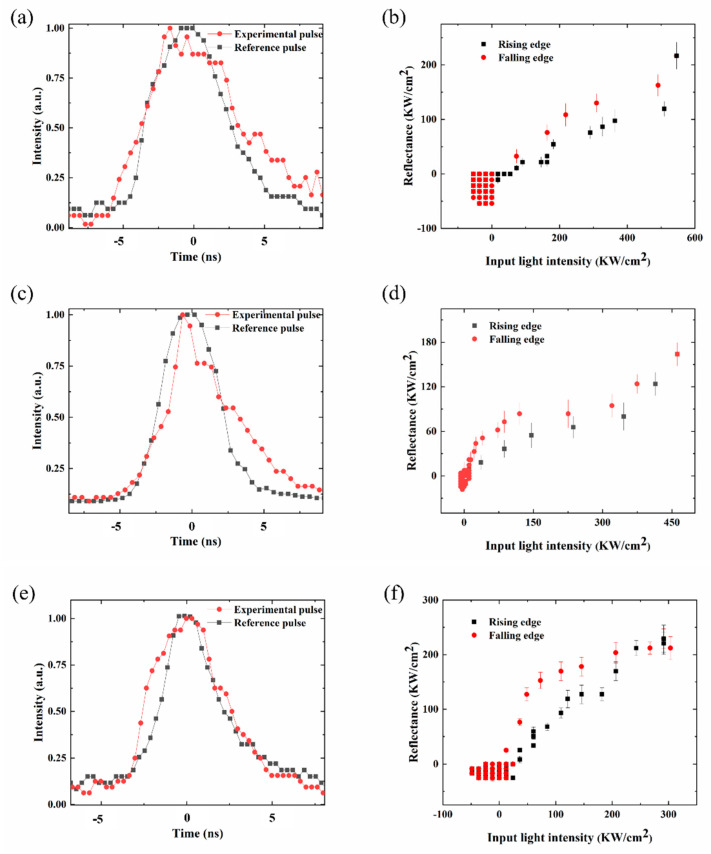
(**a**,**c**,**e**) The two waveforms displayed on the high-speed numerical oscilloscope. (**b**,**d**,**f**) The hysteresis loops of the optical bistability realized by converting the electrical signal into the optical signal, low-threshold visible light optical bistability with light intensity of 267 KW/cm^2^ can be obtained.

To experimentally demonstrate the device optical bistability behavior, the optical characterization system was built up using the laser with the wavelength of 532 nm. Figure 4 illustrates the optical path diagram of the experiment set up. A half-wave plate and Glan-laser calcite polarizer at the front of the optical path are used to adjust the optical power of the experiment. A beam of light from a nanosecond pulsed laser with a wavelength of 532 nm passes through a beam splitting system, which splits light at a ratio of 50:50. One beam illuminates the sample as the probe beam and then reflected to a photodetector, while the other beam is directly incident on the photodetector as the reference beam. The two pulsed lasers convert optical signals into electrical signals by photodetectors, and the waveforms can be received and displayed through a high bandwidth digital oscilloscope. The waveforms displayed on the high-speed numerical oscilloscope are shown in Figure 5a,c,e. Data processing is performed by importing the rising and falling edge values of the reference optical pulse signal and the probe optical pulse signal on the oscilloscope into the origin software. Since photoelectric conversion is a linear process, the electrical signals can be converted back to optical signals with a power meter. The hysteresis loop of the optical bistable is shown in Figure 5b,d,f. The black points represent the rising edge of the optical bistable, and the red points represent the falling edge of the optical bistable.

The basic issue of optical bistable device is to achieve the switching threshold as low as possible. In Figure 5b, the measured switching-up threshold for device with one layer of graphene oxide is around 100 KW/cm^2^, while the switching-down threshold of the device is around 540 KW/cm^2^. These switching threshold values are much less than those with nonlinear graphene integrated between Bragg reflectors (around 10 GW/cm^2^) [18] or two thin dielectric layers (several MW/cm^2^) [19]. This lower threshold value originates from the enhanced nonlinear absorption of graphene film integrated in our designed asymmetric nanocavity, which provides strong local field confinement, as shown in Figure 1c. In addition, the threshold values can be further reduced via increasing the graphene oxide thickness. The switching-up and switching-down threshold values are around 50 KW/cm^2^ and 414 KW/cm^2^, respectively, for three layers of graphene oxide integrated nanocavity as shown in Figure 5d, while for six-layer of graphene oxide, the switching-down threshold of the device is as low as 267 KW/cm^2^ and the switching-up threshold is only reduced to around 40 KW/cm^2^ as shown in Figure 5e. This is mainly due to that more light can be absorbed in the graphene oxide film in the nanocavity as the graphene oxide film thickness increases as illustrated in Figure 1b. The device with six layers of graphene oxide film can deliver a higher nonlinear absorption than that with only one layer of graphene oxide film, leading to the required optical bistable threshold decreased, which match quite well with the simulation results as illustrated in Figure 2. It can also be found that the graphene oxide thickness has more influence on the switching-down threshold than the switching-up threshold via the comparison of Figure 5b,d,e.

## 4. Conclusions

In conclusion, we have developed an optical bistability device by integrating graphene oxide layers into the metal-dielectric-metal asymmetric nanocavity. The nonlinear absorption of the graphene oxide in the nanocavity can be enhanced, leading to the optical bistability behavior at the wavelength of 532 nm demonstrated both by the simulation and experiment. It can also be found that with the increase of the number of graphene oxide layers, the required threshold for optical bistability decreased. For the first time, we obtained low-threshold visible light optical bistability using graphene oxide as a nonlinear material with a light intensity of 267 KW/cm^2^. The findings of this paper have important implications for realizing optical storage, optical switches, and optical amplifiers under low-threshold visible light conditions.

## Data Availability

Not applicable.
